# 1147. Regional Trends in Favipiravir and Steroid Use in the Treatment of COVID-19

**DOI:** 10.1093/ofid/ofac492.985

**Published:** 2022-12-15

**Authors:** Yusuke Asai, Shinya Tsuzuki, Nobuaki Matsunaga, Norio Ohmagari

**Affiliations:** National Center for Global Health and Medicine, Shinjuku, Tokyo, Japan; National Center for Global Health and Medicine, Shinjuku, Tokyo, Japan; National Center for Global Health and Medicine, Shinjuku, Tokyo, Japan; National Center for Global Health and Medicine, Shinjuku, Tokyo, Japan

## Abstract

**Background:**

Two years have passed since the global outbreak of COVID-19 began. Vaccines and many therapeutic agents have now been developed, and treatment is being conducted in accordance with guidelines. In general, it takes a long time for guidelines to be established, as a large amount of clinical data is required. Therefore, in the early and middle stages of an epidemic, treatment is often based on experience at individual centers. This study focuses on two drugs, Favipiravir and Steroid, and investigates how trends in drug use changed in different regions.

**Methods:**

We compared the proportion of drug administered patients in the COVID-19 Registry Japan (COVIREGI-JP). Data from four COVID-19 epidemic waves, from January 2020 to June 2021, were included for the analysis. To compare regional trends, 10 categories were used based on existing classifications. In addition, Tokyo and Osaka were accounted for separately, for a total of 12 regions. Severity of each case was divided into mild, moderate 1, moderate 2 and severe based on the condition on admission, and the proportion of Favipiravir or Steroid administered cases was calculated for moderate 2 and severe cases.

**Results:**

Favipiravir was administered to more than 50-100% of patients in the first wave. Thereafter, it declined nationwide, with sharp falls in Tokyo (34.9%, 16.5% and 4.3%) and Osaka (48.8%, 41.8% and 8.0%). In Hokkaido, on the other hand, 82.4%, 53.7% and 59.8% of the cases still continued to receive Favipiravir.

In the first wave, Steroid was administered to 20-40% of cases. The proportion gradually increased, with 50-80% in the second wave, and 85.5% in Tokyo, 93.4% in Osaka and 90.2% in Hokkaido in the fourth wave, the majority of cases.

Changes over time in the proportions of cases treated with Favipiravir and Steroid in each region.

**Figure:**
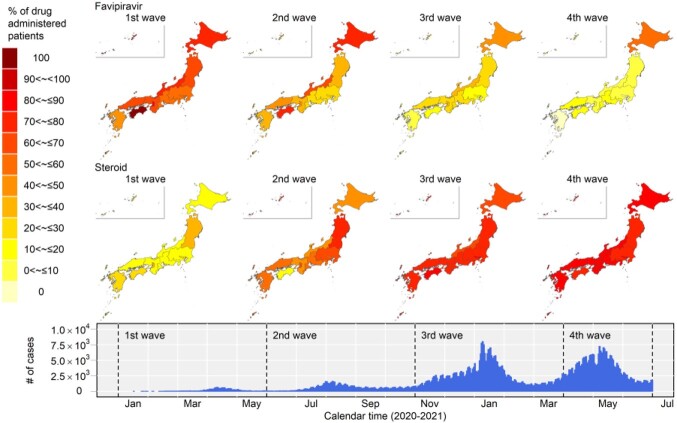

The top four and middle four panels show the proportion of cases treated with Favipiravir and Steroid, respectively. The lower epi-curve shows the number of COVID-19 cases in Japan.

**Conclusion:**

We confirmed that the more effective treatment was rapidly spreading throughout the country. More information is available in areas with a large number of cases, such as Tokyo and Osaka, and in facilities that see a large number of cases. On the other hand, it may be difficult for smaller facilities or facilities that do not see many COVID-19 cases. Information from registry studies would be useful in making more effective treatments available earlier and more widely. We believe that further use of COVIREGI-JP would promote standardization of treatment.

**Disclosures:**

**All Authors**: No reported disclosures.

